# On-chip parallel Fourier transform spectrometer for broadband selective infrared spectral sensing

**DOI:** 10.1038/s41378-019-0111-0

**Published:** 2020-02-10

**Authors:** Alaa Fathy, Yasser M. Sabry, Sébastien Nazeer, Tarik Bourouina, Diaa A. Khalil

**Affiliations:** 1 Faculty of Engineering, Ain-Shams University, 1 Elsarayat St. Abbassia, Cairo, Egypt; 2 Si-Ware Systems, 3 Khalid Ibn Al-Waleed St., Heliopolis, Cairo, Egypt; 3 Université Paris-Est, ESYCOM EA 2552, ESIEE Paris, 93162 Noisy-le-Grand, France

**Keywords:** Micro-optics, Optical sensors, Micro-optics, Chemistry

## Abstract

Optical spectrometers enable contactless chemical analysis. However, decreasing both their size and cost appears to be a prerequisite to their widespread deployment. Chip-scale implementation of optical spectrometers still requires tackling two main challenges. First, operation over a broad spectral range extending to the infrared is required to enable covering the molecular absorption spectrum of a broad variety of materials. This is addressed in our work with an Micro-Electro Mechanical Systems (MEMS)-based Fourier transform infrared spectrometer with an embedded movable micro-mirror on a silicon chip. Second, fine spectral resolution *Δλ* is also required to facilitate screening over several chemicals. A fundamental limit states that *Δλ* is inversely proportional to the mirror motion range, which cannot exceed the chip size. To boost the spectral resolution beyond this limit, we propose the concept of parallel (or multi-core) FTIR, where multiple interferometers provide complementary optical paths using the same actuator and within the same chip. The concept scalability is validated with 4 interferometers, leading to approximately 3 times better spectral resolution. After the atmospheric contents of a greenhouse gas are monitored, the methane absorption bands are successfully measured and discriminated using the presented device.

## Micro-spectrometry: chip-scale optical spectrometer for ubiquitous chemical analysis

A chip-scale optical spectrometer using a microelectromechanical system allows selective and contactless chemical analysis in the field by means of infrared spectral sensing. The widespread application of optical spectrometers for chemical analysis has been hampered by their size and cost. However, a team of scientists from Egypt and France was able to overcome the two main challenges to greater deployment of such spectrometers. First, the authors were able to extend the spectrometers’ spectral range to the infrared, thereby allowing analysis of a broad variety of materials. Second, the team could achieve fine spectral resolution, facilitating distinguishing different chemical substances. The authors plan to enhance their system to mid-infrared or even far-infrared wavelengths, and they believe that possible applications include air-quality monitoring, food analysis, precision agriculture and medical diagnosis.

Miniaturization of sensors based on MEMS technologies is now a proven option for performing measurements in low-cost and wide-scale deployments. Their integration in very large volume markets such as smartphones, cars and many other consumer products opens up new prospects for the internet-of-things (IoT). However, this success is still limited to the measurement of physical parameters only. Chemical parameters are so numerous that one can hardly consider having as many chemical sensors as chemical substances, which are quite diverse. To address this difficulty, a paradigm shift regarding implementing concepts of analytical chemistry on a silicon chip has been introduced. Although micro-gas chromatography^1^ is among the most interesting options that have been thoroughly investigated, optical spectroscopy seems to be the only way to carry out contactless, remote chemical analysis. Therefore, microscale optical spectroscopy is attracting increasing interest^2–11^. Many of the reported solutions are limited to the visible spectral range^2–4^, while those addressing the near-infrared range have a very limited spectral range^5,7,8^. In addition, very few attempts extend to the mid-infrared^9,10^, and those that do still have a very limited bandwidth. In fact, MEMS spectrometers can be based on different configurations^12^ such as diffraction gratings^13^, digital micro-mirror devices (DMDs)^11^, Fourier transform interferometers^14^, linear variable filters and tuneable Fabry-Pérot filters^15^.

Fourier transform infrared (FTIR) spectrometers appear to be a promising option since their broad spectral range enables covering the absorption molecular spectrum of a broad variety of materials, including gases, liquids and solids. In addition, a high signal-to-noise ratio (SNR) can be achieved due to the detection of all wavelengths simultaneously with a single detector^16^. In its benchtop form, it is currently used in medical analysis^17–21^, food quality control^22–25^ and soil analysis^26^, to name a few application areas. A vast majority of these applications require both high SNR and fine spectral resolution. For instance, in gas sensing, a resolution between 0.5 and 2 cm^−1^ is needed^27^. In medical applications, insulin can be analysed in the mid-IR with a resolution of 4 cm^−1^ ^28^, and the glucose concentration in blood has been determined^27^. Blood cells can be identified using FTIR with a resolution of ~2 cm^−1^ ^29^. Food quality control requires a resolution from 4 to 8 cm^−1^ ^30^, and liquids are commonly measured with a resolution ranging from 4 to 8 cm^−1^ ^19^.

Considering the miniaturization format, MEMS-based FTIR spectrometers using different architectures have already been implemented, such as the Michelson interferometer^14^, lamellar grating^31^, Mach-Zehnder interferometer^8,32^, moving wedge interferometer^33^, low finesse Fabry-Pérot interferometer^34^ and cascaded Fabry-Pérot interferometers^35^. However, all the reported solutions have a limited spectral resolution. On the one hand, the monolithic integration of all the components of the Michelson interferometer led to a resolution of ~33 cm^−1^ ^14^. On the other hand, better resolution could be achieved, but at the loss of the advantage of monolithic integration, leading to challenging alignment and high assembly costs; for instance, a resolution of ~25 cm^−1^ in the spectral range from 2 to 5 μm^36^ has been obtained with a 3-mm-diameter MEMS mirror that requires further assembly with other discrete components to form the interferometer. A moving mirror with a relatively long physical travel range of ~600 μm was assembled perpendicular to the substrate with other Michelson components and led to a resolution of ~8 cm^−1^ ^37^. In the case of the MEMS-lamellar grating spectrometer, a resolution of ~15 cm^−1^ within the mid-IR range has been achieved^31^, and gas measurement has been demonstrated with a resolution of ~20 cm^−1^ ^38^. In all FTIR spectrometers, the spectral resolution Δ*λ* is inversely proportional to the mirror travel range Δ*x*, i.e., Δ*λ* ~ 1/Δ*x*, such that achieving fine spectral resolution requires a large travel range. Therefore, the limitation of the on-chip resolution arises from the restricted travel range of the micro-mirror, limited by the stability and reliability requirements of the MEMS actuators^39^.

In this work, the parallel (or multi-core) spectrometer concept is introduced, and a silicon chip monolithically integrating multiple interferometers sharing a single MEMS actuator is implemented. This concept is intended to overcome the limitation in the spectral resolution, where the interferometers have complementary optical path differences (OPDs). The moving mirrors of the interferometers are mechanically coupled to the same actuator, while each of the *N* interferometers in the chip scans a different range of the OPD in the measured interferogram due to the complementary shifts in the mirror position implemented in the design. The interferogram of each interferometer is measured by a corresponding detector, and then signal processing is applied to produce an overall interferogram as a concatenation of the different recorded interferograms. Therefore, one can increase the OPD *N* times and thus enhance the wavelength resolution accordingly using the same MEMS actuator.

## Results

### Concept and modelling

**Fig. 1:**
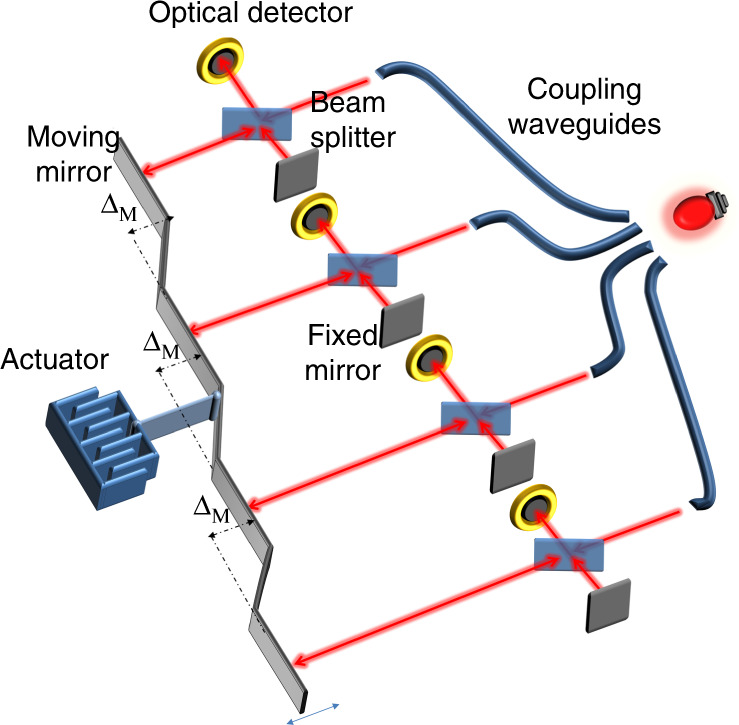
The parallel interferometers have a common actuator, where the moving mirrors are attached and spatially shifted to acquire the shifted interferograms. Each interferometer has its respective detector

An architecture consisting of four interferometers is depicted in Fig. 1. The input light is injected into the different interferometers by means of an optical fibre bundle, where the interferometers are implemented using the micro-optical bench (MOP) technology^40^. Usually, broadband thermal light sources are larger in size than the input aperture of a single MOP interferometer. For instance, the filament length is a few millimetres with a large divergence angle, while the MEMS device dimensions are hundreds of microns with a limited acceptance angle (low optical throughput). Therefore, one can easily spatially split the source power over the *N* interferometers without reducing the amount of light received by each of those interferometers. This interestingly means that the SNR per interferometer is not affected by the splitting of the light.

Each interferometer has its own beam splitter, fixed mirror, moving mirror and detector. The four moving mirrors of the four interferometers are coupled to the same actuator, while the mirror positions are shifted with respect to each other by an increment Δ_*M*_ such that the resolution can be enhanced up to four times. For instance, if the actuator travel range is 200 μm and the increment Δ_*M*_ is 200 μm, a full OPD of ~1600 μm is obtained using the four interferometers. The first interferometer scans the OPD from −800 to −400 μm, the second one scans from −400 to 0 μm, the third one scans from 0 to 400 μm and the last one scans from 400 to 800 μm. The overall interferogram is the concatenation of the four complementary interferograms, and the corresponding spectral resolution is expected to be quadrupled compared to that of a single interferometer.

### Concatenation algorithm

Due to the microfabrication non-idealities, such as variations in the over-etching in the silicon across the MEMS chip, the interferometers are not fully matched, and shifts in the OPD (along the horizontal axis of the interferograms) can be encountered with respect to the design. Additionally, the detectors used are not fully matched, leading to mismatch in the power value (along the vertical axis of the interferograms). To overcome these effects, the overlap regions between the OPDs of the different interferograms are introduced for the purpose of calibrating the concatenation positions. The moving mirror of each interferometer is designed to scan the OPD range of 2*L* + 2Δ*L*, where 2*L* is the actual optical full travel range and 2Δ*L* represents the overlap region length with respect to the interferogram of the next interferometer. The correction in the OPD depends on introducing an offset Δ*x* in the OPD of one of the two interferograms that gives the best match between the two overlapped parts of the two consecutive interferometers. The matching is calculated using Pearson’s coefficient PC, given by:

1 $$\begin{array}{l}{\mathrm {PC}}\left( {{\mathrm{\Delta }}x} \right) = \frac{{\mathop {\sum }\nolimits_i \left[\left( {I_n\left( {x_i + {\mathrm{\Delta }}x} \right)\, - \,\overline {I_n\left( {x + {\mathrm{\Delta }}x} \right)} } \right)\left( {I_{n + 1}\left( {x_i} \right)\, - \,\overline {I_{n + 1}\left( x \right)} } \right)\right]}}{{\sqrt {\mathop {\sum }\nolimits_i \left( {I_n\left( {x_i + {\mathrm{\Delta }}x} \right)\, - \,\overline {I_n\left( {x + {\mathrm{\Delta }}x} \right)} } \right)^2} \sqrt {\mathop {\sum }\nolimits_i \left( {I_{n + 1}\left( {x_i} \right)\, - \,\overline {I_{n + 1}\left( x \right)} } \right)^2} }}\end{array}$$

where *I*_*n*_ and *I*_*n*+1_ represent the intensity of the overlapped parts of interferograms of the two consecutive interferometers *n* and *n* + 1, respectively, *x*_*i*_ is the *OPD* defined in the overlapped part of the interferograms, and the upper bar represents the mean. The optimum shift Δ*x* corresponds to a maximum value for the PC.

To find the gain correction required for the interferogram power, one of the interferograms is multiplied by a variable gain, which makes the overlapped part of the interferograms nearly equal. The normalized root mean square error NRMSE is used as a merit function, where it is calculated over the overlapped region as

2 $${\mathrm {NRMSE}} = \frac{{\sqrt {\overline {\left( {I_1(x_i) - I_2(x_i)} \right)^2} } }}{{{\mathrm{max}}\left( {I_1\left( {x_i} \right)} \right)}}$$

where max represents the maximum operator. The best gain corresponds to the minimum NRMSE.

### Noise analysis

An analytical formula for the SNR can be derived in the case of additive sources of noise, such as thermal, shot and quantization noises. A noisy interferogram can be expressed by:

3 $$I\left( x \right) = I_o\left( x \right)rect\left( {\frac{x}{{2L}}} \right) + n_o\left( x \right)rect\left( {\frac{x}{{2L}}} \right)$$

where *I*_*o*_ (*x*) and *n*_*o*_ (*x*) are the desired interferogram signal and noise contribution, respectively. The MEMS average velocity is given by *V*_e_ = *L/T*_scan_, where *T*_scan_ is the scan time. Thus, the noise power can be expressed by *σ*^2^ = *N*_*O*_*B*_*f*_ = 2*N*_*o*_*V*_*e*_*B*_*v*_, where *N*_*o*_ is the noise power spectral density in *V*^2^/*Hz*, *B*_*f*_ is the electronic bandwidth of the measured spectrum in Hz and *B*_*v*_ is the corresponding wavenumber bandwidth in m^−1^. Then, the spectrum in the wavenumber domain is given by:

4 $$S\left( f \right) = \left[ {B\left( \nu \right) + N\left( \nu \right)} \right] \ast 2Lsinc\left( {2L\nu } \right)$$

where *B*(*v*) is the input (desired) spectrum, *N*(*v*) represents the noise spectrum and * represents the convolution. Then, the average noise energy density is given by^14^:

5 $$\begin{array}{l}E\left( {N\left( \nu \right)^2} \right) = \mathop {\int}\nolimits_{ - \infty }^\infty {\mathop {\int}\nolimits_{ - \infty }^\infty {rect\left({\frac{x}{{2L}}} \right)rect\left( {\frac{s}{{2L}}} \right)\sigma ^2{\it{sin}}c\left({2B_v\left( {x - s} \right)} \right)e ^{-j2\pi \left( {x - s} \right)^v}dxds} } \end{array}$$

Using the Fourier transform identities, the above expression can be rewritten as (using 1/2*L* ≪ *B*_*v*_)^41^:

6 $$E\left( {N\left( \nu \right)^2} \right) = \frac{{L\sigma ^2}}{{B_\nu }}rect\left( {\frac{\nu }{{2B_\nu }}} \right)$$

where *E* represents the expectation. Then, the *SNR* within a window Δ*v* around a central wavenumber $\bar \nu$ is given by:

7 $${\mathrm {SNR}}\left( {\bar \nu } \right) = \sqrt {N_{{\mathrm {scans}}}} \frac{{{\int \nolimits_{\bar \nu - \frac{{{\mathrm{\Delta }}\nu }}{2}}^{\bar \nu + \frac{{{\mathrm{\Delta }}\nu }}{2}}} B\left( \nu \right) \ast 2L\,sinc\left( {2L\nu } \right)d\nu }}{{{\int \nolimits_{\bar \nu - \frac{{{\mathrm{\Delta }}\nu }}{2}}^{\bar \nu + \frac{{{\mathrm{\Delta }}\nu }}{2}}} \sqrt {E\left( {N\left( \nu \right)^2} \right)} d\nu }}$$

where *N*_scans_ is the number of scans. Assuming that the spectrum of the input light is of uniform *B*_*o*_ value, and as Δ*v* → 0, the SNR is given by:

8 $${\mathrm {SNR}}\left( {\bar \nu } \right) = \frac{{B_o\sqrt {N_{{\mathrm {scans}}}} }}{{\sqrt {2N_oLV_e} }} = \frac{{B_o\sqrt {T_{{\mathrm {scan}}}N_{{\mathrm {scans}}}} }}{{\sqrt {2N_o} L}}$$

In a single-core spectrometer, increasing the resolution *N* times requires an increase in both the travel range and the actuator velocity by a factor of *N* while maintaining the same scan time of a single measurement *T*_scan_. Thus, the SNR will degrade by a factor of 1/*N*. Alternatively, one can increase the resolution using the same velocity while increasing the time scan of a single measurement *T*_scan_ by a factor of *N*. However, this means that the number of scans *N*_scans_ will be decreased by a factor of 1/*N* for the same total run time (*N*_scan_*T*_scan_). Thus, the overall SNR will degrade by a factor of 1/*N* as well for the same total run-time. For the introduced concept of a multi-core spectrometer acting simultaneously with multiple detectors, the velocity of the moving mirrors of the interferometers is not increased, and the full travel range *FTR* is scanned in the same amount of time as required by the single interferometer. Thus, the *SNR* will be degraded only by a factor of $1/\sqrt N$ while maintaining the same total run time. We call this improvement in the SNR the “parallel spectrometer advantage”.

### Experimental device

**Fig. 2:**
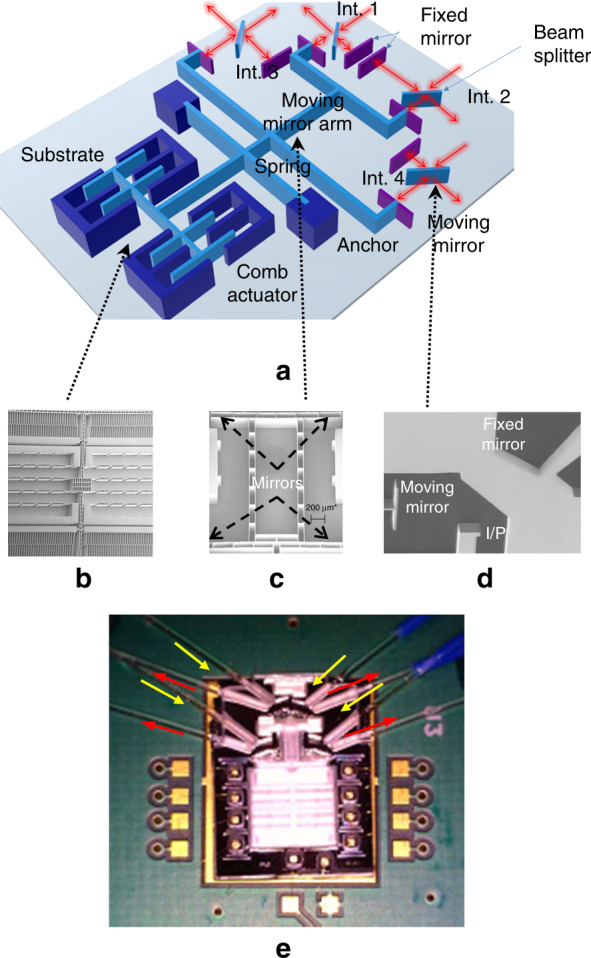
Illustration of the fabricated design of the parallel interferometers integrated on the same chip. **a** The chip-scale architecture of the MEMS spectrometer, comprising four interferometers and a comb-drive actuator. The interferometers are denoted by Int. and numbered from 1 to 4. The moving mirrors of all interferometers are attached to the same moving arm. The light path is indicated by the red arrows. **b** A SEM image of the actuator. **c** A SEM image of the moving mirror arm. **d** A SEM image of an optical interferometer. **e** A camera photo of the corresponding fabricated die with the coupling fibres, where the yellow arrows represent the input directions of the light to the interferometers and the red arrows represent the output direction of the light

The main target of the proposed design is to enhance the spectral resolution using four interferometers monolithically integrated on the same chip. This outcome was obtained by replicating the core interferometer, previously reported in ref. ^14^, designed on-chip but with shifts in the remaining positions of the moving mirrors. A three-dimensional schematic of the chip with the four interferometers is depicted in Fig. 2a. Since each of the four interferometers has a full travel range 2*L* of 360 μm, the total range is 1440 μm. Theoretically, this improves the resolution from 33 cm^−1^ to 8.3 cm^−1^, according to the full-width at half-maximum (FWHM) criterion^13^. The interferogram of the first interferometer has an OPD range from −860 to −500 μm, the second has an OPD range from −500 to −140 μm, the third has an OPD range from −140 to 220 μm, and the fourth has an OPD range from 220 to 580 μm. Thus, the centres of their OPDs corresponding to the rest positions of the moving mirrors are −680 μm, −320 μm, 40 μm and 400 μm, respectively. Additional overlap regions of the range 2Δ*L* equal to 40 μm were introduced to apply the concatenation algorithm described previously.

Scanning electron microscope (SEM) images of the fabricated comb and mirror arms are shown in Fig. 2b, c, respectively. In Fig. 2d, a SEM image of the interferometer^42^ is shown, comprising a beam splitter, a fixed mirror and a moving mirror. This design is replicated for the parallel interferometers. Regarding the quality of surfaces, the root mean square (RMS) value of the roughness is <20 nm using this technology^43^, and the measured verticality of the surfaces is better than 0.05°. The mirror reflectivity is greater than 90%. A photo of the fabricated chip assembled on a PCB daughter board is shown in Fig. 2e. The light from the optical source was coupled into the interferometers using a bundle of multimode fibres (MMFs) connected directly to the light source. On the other hand, the optical fibres were cleaved and inserted into the MEMS chip in micromachined grooves self-aligned with the interferometers. The MEMS actuator is operated at resonance using an actuation voltage with a sinusoidal waveform. It has a maximum displacement of ~250 μm. The position of the MEMS actuator was determined by means of a capacitive sensing circuit. The relation between the mirror position and the capacitance was calibrated using a reference light at 1550 nm^44^.

The output optical light beams from the interferometers are different, they cannot be summed on the same detector, and the output of each one should be coupled to its corresponding detector. The output electrical signal from the corresponding detector is amplified by electronic amplifiers. These are followed by an analog-to-digital converter (ADC) to sample the analog signal and convert it to the digital domain, where digital filtering is applied to obtain the corresponding interferogram. After time averaging of each of the four interferograms, the concatenation algorithm is applied, and the final spectrum is obtained. The scan time *T*_scan_ is ~1.5 ms, while *N*_scans_ is 1000 scans or more.

### White light characterization

**Fig. 3:**
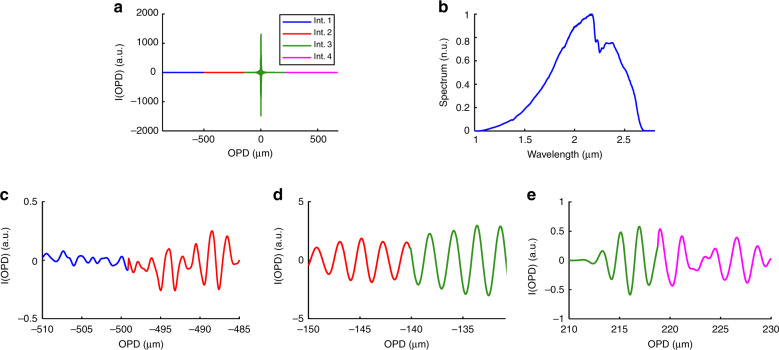
White light measurement using the parallel interferometers. **a** The concatenated interferograms, where the interferogram of each interferometer, denoted as Int., is drawn with different colours. **b** The corresponding spectrum of the concatenated interferograms. **c** Intersecting part of the spectrum of Int. 1 and Int. 2. **d** Intersecting part of the spectrum of Int. 2 and Int. 3. **e** Intersecting part of the spectrum of Int. 3 and Int. 4

A white light source was measured by the four interferometers, where each one had its own interferogram versus the respective OPD. The overall interferogram after concatenation is shown in Fig. 3a, where the interferogram comprises four parts of different colours corresponding to the four different interferograms. Applying the Fourier transform to the concatenated interferograms gives the corresponding spectrum of higher resolution, as depicted in Fig. 3b. The intersecting part of the interferograms of interferometers 1 and 2 is shown in Fig. 3c, the intersecting part of the interferograms of interferometers 2 and 3 is shown in Fig. 3d, and the intersecting part of the interferograms of interferometers 3 and 4 is shown in Fig. 3e. The intersection of the interferograms of interferometers 2 and 3 and that of interferometers 3 and 4 are smoother than those of interferometers 1 and 2 because the signal power in these parts is higher and, thus, the noise effect in the transitions is smaller.

### Characterization with narrow band filters

**Fig. 4:**
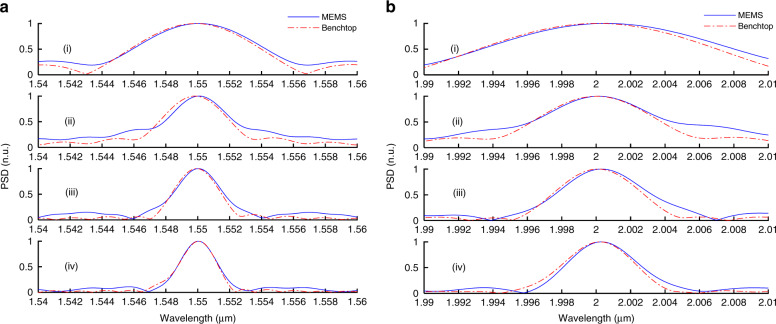
Spectra of narrow band sources (white light followed by narrow band filters) measured by the parallel spectrometer design (blue bold) and by the benchtop spectrometer for the same corresponding OPD range (red dash dot). i Spectrum of a single interferometer. ii, iii and iv Spectrum of the concatenated interferograms of two interferometers, three interferometers and four interferometers, respectively. **a** Spectra of the filter at 1.55 μm. **b** Spectra of the filter at 2 μm

To test the resolution enhancement achieved by the parallel interferometers, an optical filter with an FWHM of 2 nm cantered at 1.55 μm was placed after the white light lamp to obtain a narrow band spectrum. Additionally, the same filter was measured using a benchtop spectrometer (Bruker Tensor II). The spectrum obtained from a single interferometer, which contains the main burst, is shown in Fig. 4a(i) (depicted by the blue bold line) and compared with a spectrum measured with the benchtop spectrometer for the same corresponding OPD range (depicted by the red dash-dot line). The spectra of the concatenated interferograms of two consecutive interferometers, three consecutive interferometers and four consecutive interferometers are shown in Fig. 4a(i), (ii) and (iv), respectively. The figures show the gradual improvement of the resolution of the spectrum with increasing number of concatenated interferograms. The measured FWHM was improved from 8.4 nm to 2.7 nm (from 35 cm^−1^ to 11 cm^−1^). Moreover, another filter centred at 2 μm with an FWHM of 1 nm was also measured using the MEMS interferometers and the benchtop spectrometer. The obtained spectra are depicted in Fig. 4b. The measured FWHM was 4.9 nm, obtained using the four interferometers. The corresponding value obtained using a single interferometer was 14.8 nm. At both wavelengths, the improvement in the resolution is ~3.1-fold, which is smaller than the ideal expected fourfold improvement. This result is due to the self-apodization of the interferogram, which affects the achieved resolution^16^.

### Krypton source spectral analysis

**Fig. 5:**
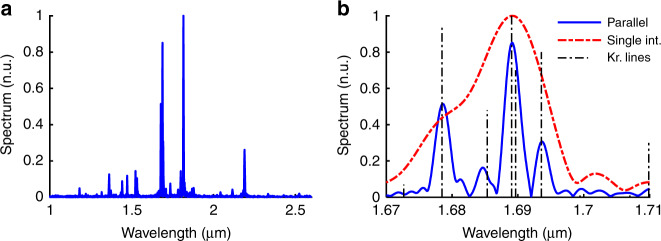
Measured spectrum of the krypton source. **a** Krypton emission spectrum measured by the parallel interferometers. **b** Zoomed-in image of the wavelength range of 1.67–1.71 μm. The measured krypton peaks in the case of the four parallel interferometers (in blue) and single interferometer (in red) are shown. Reference lines of krypton are also plotted (dashed black lines)

A krypton source is characterized by having distributed emission lines in the infrared region. The emission spectrum of this source was measured using a single interferometer and parallel interferometers. The measured spectra around a wavelength of 1.69 μm are plotted in Fig. 5. For reference, the krypton emission lines taken from the literature^45^ are also shown (dashed black lines). The spectrum obtained by the single interferometer (red curve) does not discriminate the emission lines at 1.678 μm and 1.688 μm due to the limited spectral resolution. Using the parallel interferometers (blue curve), these emission lines are discriminated successfully.

### Methane gas spectral analysis

**Fig. 6:**
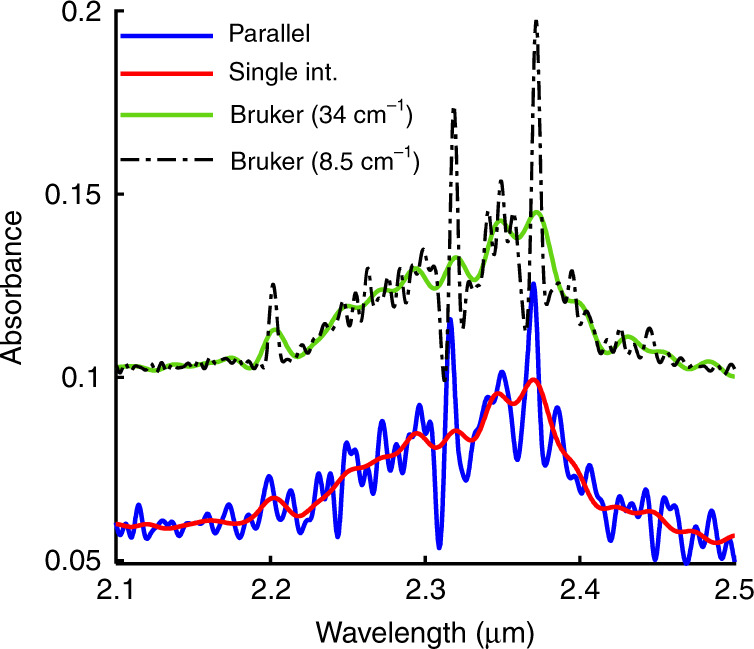
Measured absorbance spectra of methane. Measured absorbance curves of methane measured by the parallel interferometers (in blue) and the single interferometer (in red) and compared to the absorbance measured by the benchtop spectrometer for the two corresponding resolutions

A gas measurement was also conducted to show the ability of the parallel interferometers to discriminate gas absorption bands. A gas cell that contains 10% methane (CH_4_) was inserted between the white light source and the spectrometer. The gas cell length is 10 cm. The content of the gas cell was analysed using parallel interferometers and a single interferometer. The obtained absorbance curves are shown in Fig. 6. The absorption bands around 2.32 μm and 2.37 μm are apparently discriminated (blue curve) compared to the spectrum given by the single interferometer (red curve). The absorbance value was also enhanced dramatically, as shown by the wavelength range of 2.3–2.37 μm. For reference, the same gas cell was also measured using the benchtop spectrometer for two different resolutions, namely, 34 cm^−1^ and 8.5 cm^−1^, and plotted in Fig. 6. These resolutions are the theoretical resolutions of the single MEMS interferometer and the MEMS parallel interferometers. The comparison shows good agreement with the measurements of the MEMS spectrometer.

### Signal-to-noise ratio

**Fig. 7:**
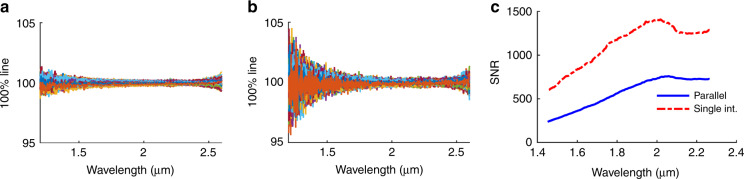
Signal-to-noise ratio of the new architecture compared to the single interferometer. **a** 100% line versus wavelength of a single interferometer. **b** 100% line versus wavelength of parallel interferometers. **c** Corresponding SNR versus wavelength in the case of parallel interferometers and a single interferometer

To test the SNR of the proposed spectrometer architecture and compare the results with the derived theoretical predictions, the SNR was measured using the 100%-line method^16^ for the single interferometer and the parallel interferometers. The method is based on measuring successive spectra and dividing every two consecutive spectra to obtain a group of 100% lines. The measurement setup was in transmission mode. The measurement time was 2 s. The 100% lines of the single interferometer and those of the concatenated interferograms are shown in Fig. 7a, b, respectively. The noise is increased due to the increased OPD. The SNR versus wavelength in the case of the parallel interferometers and the single interferometer (Int. 3) is shown in Fig. 7c. The ratio between the SNR of the parallel interferometers and that of the single interferometer ranges from 0.35 to 0.6 across the wavelength range, with a mean value of ~0.5, as expected from Eq. (8). The peak value of the SNR of the MEMS parallel interferometer MEMS is ~760:1 at 2.058 μm. This result corresponds to an RMS value of 0.13%, which is lower than the standard required value of 0.2% stability over 2 h.

## Discussion

The proposed parallel spectrometer architecture is eventually proven to allow going beyond the fundamental limit of the conventional FTIR spectrometer, the wavelength resolution Δ*λ* of which is known to be inversely proportional to the travel range Δ*x* of the movable mirror. This is a significant limitation when considering the chip-scale spectrometers, since Δ*x* is limited by the chip size, which is a few millimetres.

The practical implementation of the concept of parallel FTIR spectrometers was achieved in this work based on standard MEMS technologies on a centimetre-scale chip. The proposed architecture schematically depicted in Figs. 1 and 2 is scalable. It has the advantage of sharing the same actuator for simultaneously acting on the shifting motions of all movable mirrors of the *N* Michelson interferometers, the individual footprint of which did not exceed a few mm^2^. To validate the predicted performance scalability rules, a MEMS die comprising four parallel interferometers was fabricated and tested. The achieved spectral resolution was 11 cm^−1^, compared to 35 cm^−1^ for a single interferometer. The corresponding improvement of Δ*λ* is 3.1-fold. The proposed structure also did not sacrifice the input power because the thermal infrared light source area is much larger than that of each single MEMS interferometer. Here is the origin of the idea to use multiple interferometers on the same chip to make use of the large source area while simultaneously achieving higher resolution. In this scheme, the SNR was degraded by only 0.5 times while maintaining the same total run time, in agreement with our theoretical modelling, which predicted a scaling as ${\mathrm {SNR}}\sim 1/\sqrt N$. Furthermore, based on its improved resolution, it was shown that the proposed device is capable of recognizing the spectral signature of methane through several of its characteristic lines. The proposed structure used eight optical fibres for light coupling in and out of the interferometers, which adds some complexity to the system. In principle, the fibres can be replaced by waveguides monolithically integrated on the same chip and a micro-optical coupling component^46,47^ to achieve the ultimate goal of compactness and low cost.

Based on these first results of spectral sensing, as well as the experimental validation of the scalability rules established in theoretical modelling, this work opens the way for selective and contactless chemical analysis in-the-field, based on the chip-scale FTIR-MEMS optical spectrometers, especially when one considers the possible wavelength range that can be extended further to the mid-infrared and even far-infrared. Typical target applications include environmental monitoring, including air quality and soil analysis, precision agriculture and medical diagnosis.

## Methods

### Fabrication

The chip was fabricated using MEMS technology. The chip size was 9.1 × 12 mm^2^. A silicon-on-insulator (SOI) wafer with a device layer of 400 μm and oxide thickness of 3 μm was used. The deep reactive ion etching (DRIE) process is used to etch the deep trenches to achieve a high-throughput interferometer. Smooth surfaces were achieved using post-oxidation. Metallization was further applied for mirrors and pads. More details on the process steps can be found in the Supplementary Data.

### Optical measurements

For coupling light into/out of the interferometers, four pairs of MMFs (Thorlabs) were used. The core/cladding diameter was equal to 400/440 μm, and the numerical aperture (NA) was equal to 0.39. Each interferometer has its perspective input and output grooves for placing the fibre inside. Different light sources were used for light injection into the interferometers. For a wide spectrum, a halogen lamp (Avantes Avalight) was used. The source temperature was 3000 K. Multilayer Bragg filters (Omega optics; one with an FWHM of 2 nm at 1.55 μm and the other with an FWHM of 1 nm at 2 μm) were used to narrow the wide light coming from the halogen light source for resolution measurements. The filter FWHM is 2 nm. A krypton source (Ocean optics Kr-1) was used as the multi-wavelength emission source. For the output interferogram detection, extended InGaAs detectors (1.1–2.6 μm) (Hamamatsu G12183) were used for detection. To measure gases using the proposed spectrometer architecture, a standard gas cell (Wavelength references, Inc.) containing 10% methane, 1% acetylene, 0.25% carbon dioxide and 4% carbon monoxide diluted in nitrogen was used. The total pressure inside the cell was 740 Torr. The cell was placed between the light source and the parallel interferometer chip. The gas length was 10 cm. Methane was the most dominant gas absorbed in the spectral range of the spectrometer. Collimation lenses were added at the ends of the gas cell. A benchtop spectrometer (Bruker Tensor II) was used to validate the results of the proposed MEMS spectrometer.

## Supplementary information

Supplementary_data

## Data Availability

All published data supporting the findings of this study are available from the corresponding author upon reasonable request.
