# Supplementary material for: On-chip parallel Fourier transform spectrometer for broadband selective infrared spectral sensing
Source: Microsyst Nanoeng. 2020 Feb 10;6:10. doi: 10.1038/s41378-019-0111-0 (PMC8433235; doi:10.1038/s41378-019-0111-0)
Supplement: Supplementary file 1 — Supplementary_data [file 41378_2019_111_MOESM1_ESM.pdf]

On-chip parallel Fourier transform spectrometer for broadband selective infrared spectral sensing

Supplementary Information

Alaa Fathy[1,2,3], Yasser M. Sabry[1,2], Sébastien Nazeer [2], *Tarik Bourouina*[2,3,*] and Diaa A. Khalil[1,2]

[1]Faculty of Engineering, Ain-Shams University, 1 Elsarayat St. Abbassia, Cairo, Egypt.
[2]Si-Ware Systems, 3 Khalid Ibn Al-Waleed St., Heliopolis, Cairo, Egypt.
[3]Université Paris-Est, ESYCOM EA 2552, ESIEE Paris – 93162 Noisy-le-Grand, France.

* Corresponding author: *Tarik Bourouina* (tarik.bourouina@esiee.fr)

Fabrication process flow

The MEMS fabrication process sequence is schematically depicted in Figure S1. First, an oxide layer is formed on the device layer by oxidation as depicted in Figure S1 (a). A photo resist layer is spin-coated on the device layer and patterned using photolithographic mask according to the design as depicted in Figure S1 (b). In Figure S1 (c), the oxide layer is etched using the DRIE process. The oxide layer becomes the mask for the DRIE of the device layer as depicted in Figure S1 (d). The DRIE process is followed by oxidation process to smooth the surfaces and, consequently, the roughness scalloping size is decreased. Then the BOX layer is etched using vaporized hydrofluoric acid (vapor HF) and the moving structures are released as shown in Figure S1 (e). Finally, metal is sputtered using a shadow mask as shown in Figure S1 (f) to form the pads and the mirrors.

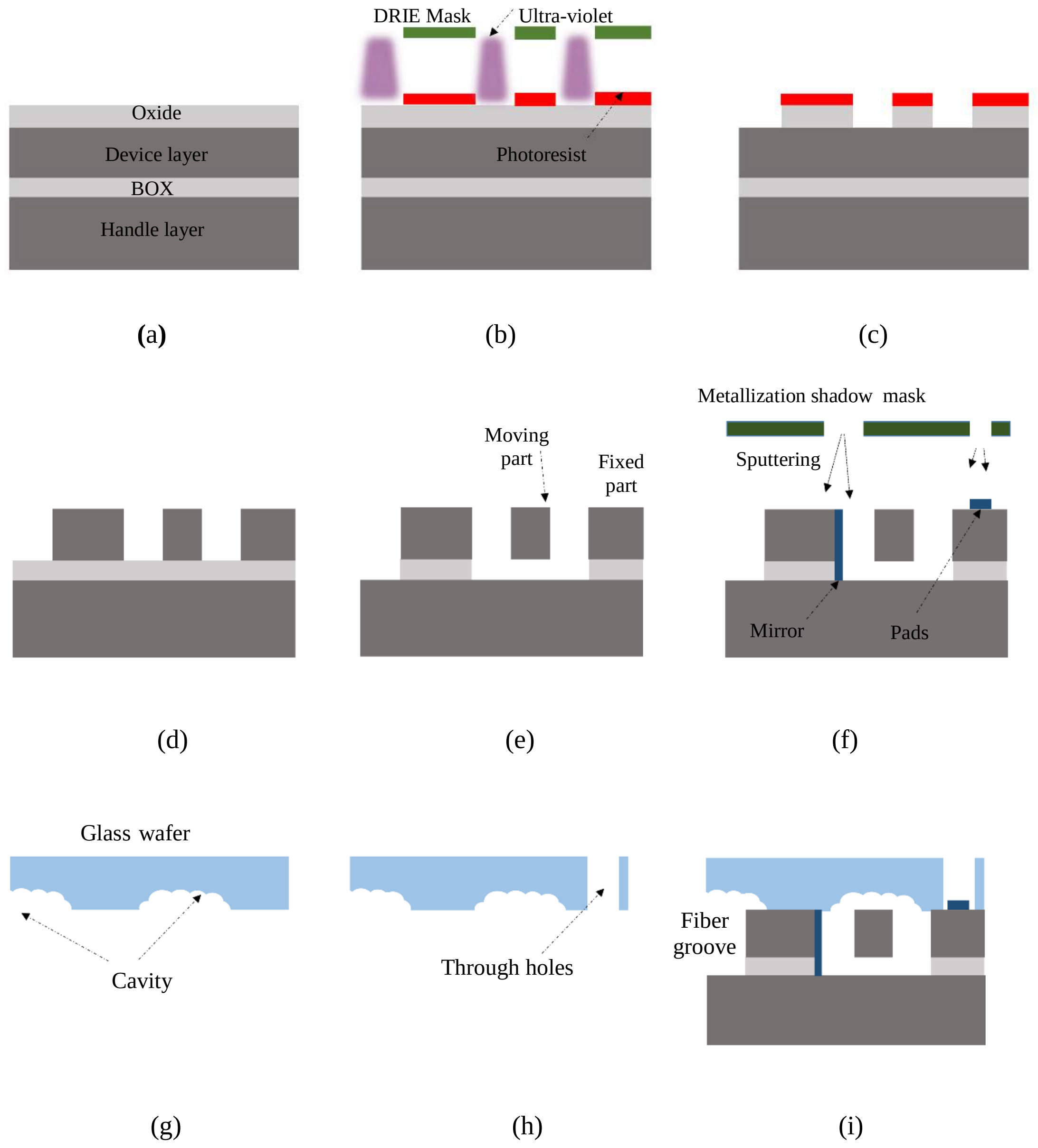

Figure S1. MEMS fabrication flow (a) Device layer oxidation. (b) Photolithography. (c) Device oxide etching. (d) Device layer DRIE. (e) BOX etching to release structures. (f) Metallization. (g) Cavity etching in the bottom of glass wafer. (h) Through holes etching in the glass wafer. (d) Anodic bonding of glass wafer and SOI wafer.

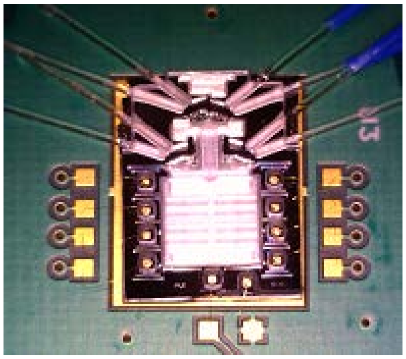

Figure S2 MEMS FTIR interferometer chip

After fabricating the SOI wafer. The chip needed to be covered by a glass for protection of the moving parts and the optical interfaces. Cavity etching in the bottom of the glass wafer is needed in the regions above the actuator (to free space for the actuator so that it can move freely without hitting the cover) or above the fiber grooves (to be able to insert the fibers). This is for sure applied using a respective mask, as depicted in Figure S1 (g). The glass etching is performed by sandblasting. Sand is abrasive and when mixed with fast air causes etching and yields frosted glass as depicted by the rough cavities. Such cavity appears hazy shown in Figure S2. in the regions above the MEMS actuator and the fiber grooves. Through holes are etched in the glass wafer for pads wire bonding as depicted in Figure S1 (h). After that, the glass wafer is bonded to the fabricated SOI wafer using anodic bonding, as depicted in Figure S1 (i). The last step in the fabrication process is dicing to obtain single dies. Finally, the die is bonded to a printed circuit board PCB and wire bonding is applied to connect between the pads on the die and the copper tracks on the PCB.
